# Insights into the Synergistic Effect and Inhibition Mechanism of Composite Conditioner on Sulfur-Containing Gases during Sewage Sludge Pyrolysis

**DOI:** 10.3390/molecules29174110

**Published:** 2024-08-29

**Authors:** Shan Cheng, Lianghui Chen, Shaoshuo Wang, Kehui Yao, Hong Tian

**Affiliations:** School of Energy and Power Engineering, Changsha University of Science and Technology, Changsha 410114, China; shancheng@csust.edu.cn (S.C.);

**Keywords:** sewage sludge, pyrolysis, sulfur-containing gases, composite conditioning, synergistic interactions

## Abstract

Sewage sludge odorous gas release is a key barrier to resource utilization, and conditioners can mitigate the release of sulfur-containing gases. The gas release characteristics and sulfur compound distribution in pyrolysis products under both single and composite conditioning strategies of CaO, Fe_2_O_3_, and FeCl_3_ were investigated. This study focused on the inhibition mechanisms of these conditioners on sulfur-containing gas emissions and compared the theoretical and experimental sulfur content in the products to evaluate the potential synergistic effects of the composite conditioners. The findings indicated that at 650 °C, CaO, Fe_2_O_3_, and FeCl_3_ inhibited H_2_S release by 35.8%, 23.2%, and 9.1%, respectively. Notably, the composite of CaO with FeCl_3_ at temperatures ranging from 350 to 450 °C and the combination of Fe_2_O_3_ with FeCl_3_ at 650 °C were found to exert synergistic suppression on H_2_S emissions. The strongly alkaline CaO inhibited the metathesis reaction between HCl, a decomposition product of FeCl_3_, and the sulfur-containing compounds within the sewage sludge, thereby exerting a synergistic suppression on the emission of H_2_S. Conversely, at temperatures exceeding 550 °C, the formation of Ca-Fe compounds, such as FeCa_2_O_4_, appeared to diminish the sulfur-fixing capacity of the conditioners, resulting in increased H_2_S emissions. For instance, the combination of CaO and FeCl_3_ at 450 °C was found to synergistically reduce H_2_S emissions by 56.3%, while the combination of CaO and Fe_2_O_3_ at 650 °C synergistically enhances the release of H_2_S by 23.6%. The insights gained from this study are instrumental in optimizing the pyrolysis of sewage sludge, aiming to minimize its environmental footprint and enhance the efficiency of resource recovery.

## 1. Introduction

Sewage sludge, referred to as ‘sludge’ hereinafter, is characterized by its high water content and rich organic matter [1]. Sludge pyrolysis technology involves the thermal chemical transformation of sludge through heating under a high-temperature, inert atmosphere, which yields products such as char, tar, and non-condensable gases [2]. Pyrolysis not only facilitates the reduction in and harmless treatment of sludge, but also can obtain fuel or high-value carbon-containing materials, such as adsorbents or soil amendments [3]. During the pyrolysis process, various factors influence the generation and characteristics of the products, with temperature being one of the key factors [2]. Generally, an increase in temperature leads to a reduced yield of char and tar, while simultaneously leading to an increased yield of gaseous products. Additionally, the residence time of the sludge at the target pyrolysis temperature is an important parameter that affects the secondary reactions of tar and gas yield, as well as the specific surface area of the char [4].

The pyrolysis of sludge is accompanied by the emission of numerous odorous sulfur-containing gases, including H_2_S, SO_2_, CH_3_SH, CS_2_, and COS [1,5]. These compounds are particularly concerning due to their low olfactory detection thresholds, H_2_S, for instance, can be detected at concentrations as low as 0.00041 mg/kg. The presence of such gases can have a profound negative impact on both human health and ecological systems [4,6,7]. The release of these noxious odors during sludge pyrolysis has become a significant impediment to the advancement of pyrolysis technologies [8]. Consequently, there is an urgent need for effective management of these sulfurous odorous emissions to facilitate the resourceful utilization of sludge [9].

A substantial residue of dewatering conditioners, such as Fe-based and Ca-based conditioners, remains within the sludge. The introduction of these conditioners significantly influences the emission of sulfur-containing gases during the sludge pyrolysis process. Kwon et al. observed that CaCO_3_ decomposes to CaO at high temperatures, which can effectively capture sulfurous gases [10]. This finding is supported by Cao et al., who showed that CaO can immobilize over 70% of sulfur in the form of inorganic sulfates and sulfides within the char [11]. Concurrently, Liu et al. noted a reduction in H_2_S emissions with the addition of FeCl_3_, particularly at pyrolysis temperatures between approximately 600 and 800 °C, yet an unexpected increase was observed when the temperature was further increased to 800~950 °C [12]. This suggests that the effects of various conditioners on the process are temperature-dependent and warrant additional investigation to elucidate the underlying mechanisms.

Composite conditioners have the potential to more effectively control the release of sulfurous gas emissions during sludge pyrolysis. Although a limited number of studies have explored the effects of composite conditioners, they have suggested possible synergistic effects. For instance, Liu et al. demonstrated that the concurrent addition of Fenton’s reagent and CaO is beneficial for the retention of greater sulfur content in the char during pyrolysis [13]. However, the synergistic mechanism behind this phenomenon remains unexplained. Additionally, Li et al. found that at 300 °C, a composite of Ca(OH)_2_ and Fe_2_O_3_ positively contributes to the fixation of SO_2_ emitted from pyrolysis [6]. Nonetheless, a quantitative evaluation of the synergistic effect was not performed. Therefore, it is necessary to conduct a quantitative assessment of the synergistic effects of composite conditioners and to thoroughly investigate their mechanisms in sulfur transformation. This will help advance the development of sludge treatment technologies, leading to resource utilization and causing the harmlessness of sludge.

In response, the present study employs commonly used inorganic conditioners CaO, Fe_2_O_3_, and FeCl_3_. These are blended in ratios of 1:1, 2:1, and 1:2 to form the composite conditioners. By contrasting the theoretical and actual emissions of sulfur-containing gases throughout the pyrolysis of composite-conditioned sludge at various temperatures, a quantitative analysis of the composites’ synergistic impacts is conducted. Additionally, the distribution of sulfurous substances in pyrolysis products is examined, with a particular focus on the sulfur forms in solid residues, to elucidate the synergistic mechanisms of the composite conditioning agents. The findings of this study provide a theoretical foundation for the efficient, cost-effective, and resource-orientated utilization of sludge.

## 2. Results and Discussion

### 2.1. Sulfur Distribution in Products

#### 2.1.1. Sulfur Balance

The distribution of sulfur within the products derived from raw sludge (RS) and conditioned sludge is depicted in Table 1, which is similar to previous studies showing that sulfur-containing substances were not detected in tar [14]. It is apparent that a significant portion of sulfur was retained in char, with 79.1% of sulfur being found in the RS-char and 15.1% existing in the gaseous phase. In comparison to RS, the proportion of char-S increased, and gas-S reduced in conditioned sludge. Notably, RS-CaO exhibited an 11.5% increase in char-S and an 8.1% decrease in gas-S. The application of Fe-based conditioners, particularly Fe_2_O_3_, similar to CaO, resulted in a 10.7% increase in char-S content for RS-Fe_2_O_3_ and a 6.4% increase for RS-FeCl_3_, while the gas-S was diminished by 8.2% and 5.2%, respectively. Overall, these results indicate that the three types of conditioners are effective in enhancing the sequestration of sulfur in char and reducing its release into the gaseous state.

#### 2.1.2. Release of Sulfur-Containing Gases 

**Figure 1:**
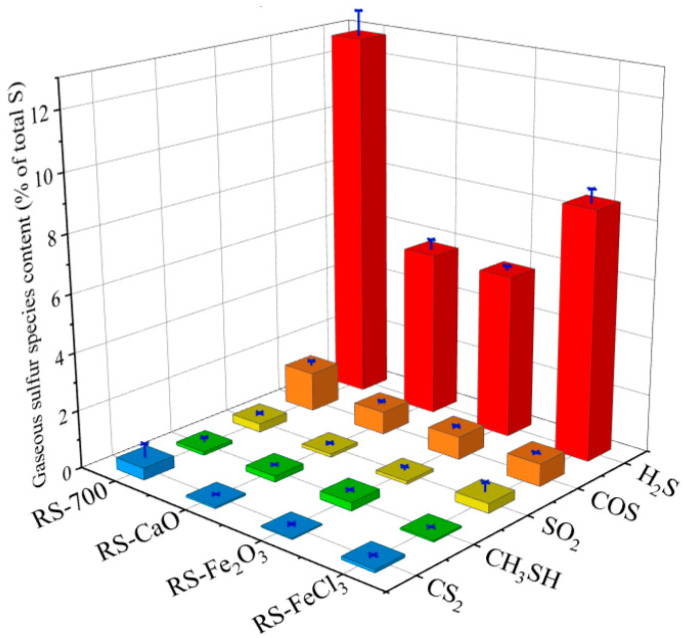
Distribution of sulfur in gaseous products. (Red represents H_2_S, orange represents COS, yellow represents SO_2_, green represents CH_3_SH, and blue represents CS_2_).

To investigate the impact of conditioners on the emission of sulfur-containing gases throughout sludge pyrolysis, a comprehensive analysis was performed to identify and quantify these gases. Figure 1 illustrates that the pyrolysis of RS primarily results in the release of H_2_S, which constitutes 85.3% of the total sulfur-containing gases emitted, followed by COS, CS_2_, SO_2_, and CH_3_SH in terms of abundance. It is hypothesized that H_2_S originates from the desulfurization of both aliphatic and aromatic sulfur compounds, COS is likely formed through the interaction of C=O with SH or via the reaction between CO_2_ and H_2_S [15]. The increase in CS_2_ is connected to the decomposition of COS, and the release of SO_2_ is linked to the oxidation of organic sulfur species, whereas the formation of CH_3_SH is attributed to secondary reactions taking place within the char matrix [16,17].

The three conditioners significantly suppressed the emission of H_2_S. Compared to RS, the addition of these conditioners led to a reduction of H_2_S release, ranging from 33 to 56%. Variations in the release of other sulfur-containing gases were observed; however, these alterations represented less than 1.3% of the total sulfur in RS. This indicates that H_2_S is more likely to be produced during sludge pyrolysis, and other types of sulfur-containing gases are more difficult to produce through secondary reactions, which is related to the higher H/C ratio of sludge compared with coal [16,17]. Given that the decrease in H_2_S for RS-CaO, RS-Fe_2_O_3_, and RS-FeCl_3_ is nearly mirrored by the increase in char-S, as depicted in Table 1, it is viable to discuss the impact of the conditioners with a focus on H_2_S. A comparison of the effects of different conditioners reveals that, following the pyrolysis of RS-CaO, the release of H_2_S constituted 5.7% of the total sludge sulfur, signifying a 55.3% reduction compared to RS. This reduction is likely due to the inhibition of desulfurization reactions involving aliphatic and aromatic sulfur compounds by CaO, or the capture of the acidic gas due to the strong alkalinity of CaO [14]. Fe-based conditioners, such as Fe_2_O_3_ and FeCl_3_, exhibited similar effects, leading to a 56.1% and 32.7% decrease in H_2_S release, respectively. This is due to the effects of Fe-based conditioners being analogous to that of CaO, which can inhibit the decomposition of aliphatic and aromatic sulfur species or capture gas-S [18,19]. Based on the preceding analysis, sulfur in the products is present as char-S and gas-S, with H_2_S being the predominant sulfur-containing gas. Consequently, subsequent studies concentrated on the release variations of H_2_S and the content changes of different sulfur forms in the solid products, thereby facilitating an in-depth analysis of the synergistic effects and the underlying mechanisms of the conditioners.

### 2.2. Effect of Conditioners on H_2_S Release

#### 2.2.1. Effect of Single Conditioner

**Figure 2:**
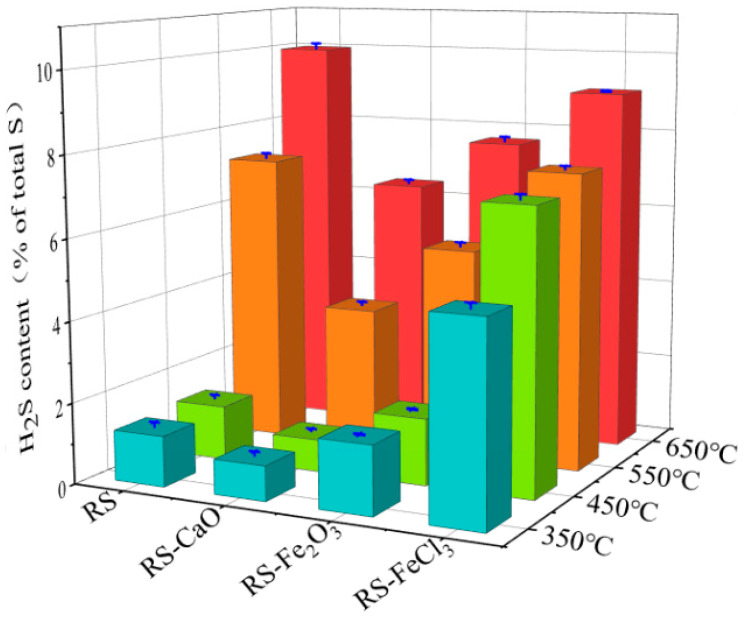
H_2_S release at various pyrolysis temperatures. (Red represent 650 °C, orange represent 550 °C, green represent 450 °C, and blue represent 350 °C).

The effects of CaO, Fe_2_O_3_, and FeCl_3_ on H_2_S emissions during the sludge pyrolysis at various temperatures are depicted in Figure 2. As the temperature increased from 350 °C to 650 °C, the emission of H_2_S from RS gradually increased from 1.3% to 10%. This phenomenon is attributed to the exacerbated decomposition of aliphatic and aromatic sulfur-containing compounds within the sludge as the temperature elevates [18]. Notably, between 450 °C and 550 °C, the concentration of H_2_S experienced a fivefold surge, rising from 1.4% to 7.3%. This observation suggests that at 550 °C, there significant sulfur decomposition within the RS, resulting in a pronounced enhancement in H_2_S yield.

For sludge amended with conditioners, CaO consistently reduced the emission of H_2_S across the temperature range of 350~650 °C, exhibiting an inhibitory effect over a broad temperature interval. In contrast, Fe_2_O_3_ exerted an inhibitory impact on H_2_S within the temperature range of 550~650 °C, while FeCl_3_ manifested a positive influence exclusively at 650 °C. These observations suggest that the underlying mechanisms of their inhibitory actions varied, thereby conferring distinct suppressive or promotional effects on H_2_S emissions at specific temperatures. Detailed discussion is as follows:

CaO exhibited the most effective suppression of H_2_S emissions, achieving a reduction between 31.2% and 51.3% relative to RS. In comparison, the inhibitory effects of Fe_2_O_3_ and FeCl_3_ were less significant. At temperatures ranging from approximately 550 to 650 °C, Fe_2_O_3_ diminished H_2_S release by 27.3% and 23.2%, respectively, while FeCl_3_ only exhibited a 9.1% reduction at 650 °C. The strong alkalinity of CaO is likely responsible for the direct capture of acidic gases such as H_2_S, leading to the formation of stable calcium–sulfur compounds. These compounds immobilize sulfur within the char and reduce the release of sulfur in gaseous form [11]. Furthermore, the oxidizing nature of CaO can promote the transformation of organic sulfur compounds such as aliphatic sulfur and aromatic sulfur into more stable sulfoxides and sulfones [20]. On the other hand, Fe_2_O_3_, with its attenuated alkaline properties, is likely to immobilize sulfur primarily through the formation of stable sulfur-containing substances at higher temperatures. The effectiveness of FeCl_3_ at high temperatures may be ascribed to its transformation into Fe_2_O_3_.

Within the temperature range from 350 to 450 °C, both Fe_2_O_3_ and FeCl_3_ demonstrated a promotional effect on the emission of H_2_S, with a particular emphasis on FeCl_3_. This phenomenon may stem from the catalytic properties of Fe, which may lower the activation energy required for the decomposition of organic sulfur compounds, thereby reducing its decomposition temperature [12]. According to Hu et al., the activation energy was reduced by 7.38% during the co-pyrolysis process due to the presence of Fe [21]. Furthermore, the role of Cl is of greater significance in enhancing the release of sulfur-containing gases. For instance, the decomposition of FeCl_3_ can yield HCl (reaction1), which may react with stable inorganic sulfides, leading to the formation of chlorides and the liberation of H_2_S [15].

(1) $${\mathrm{FeCl}}_{3}+H_{2}O\to \mathrm{Fe}{\left(\mathrm{OH}\right)}_{3}+\mathrm{HCl}\to {\mathrm{Fe}}_{2}O_{3}+H_{2}O+\mathrm{HCl}$$

#### 2.2.2. Synergistic Effect of Composite Conditioner

**Figure 3:**
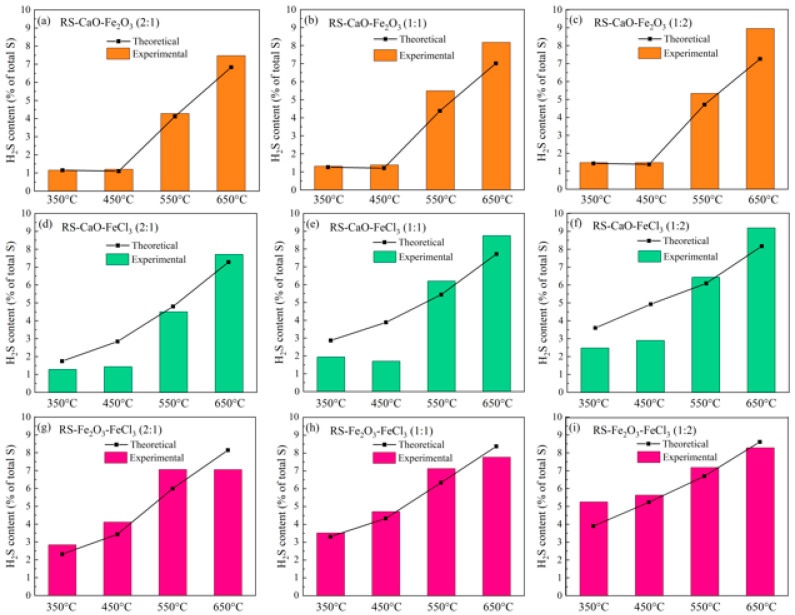
The theoretical and experimental release of H_2_S from composite-conditioned sludge: (**a**) RS-CaO-Fe_2_O_3_ (2:1), (**b**) RS-CaO-Fe_2_O_3_ (1:1), (**c**) RS-CaO-Fe_2_O_3_ (1:2), (**d**) RS-CaO-FeCl_3_ (2:1), (**e**) RS-CaO-FeCl_3_ (1:1), (**f**) RS-CaO-FeCl_3_ (1:2), (**g**) RS-Fe_2_O_3_-FeCl_3_ (2:1), (**h**) RS-Fe_2_O_3_-FeCl_3_ (1:1), and (**i**) RS-Fe_2_O_3_-FeCl_3_ (1:2).

The theoretical emission of H_2_S throughout the pyrolysis of composite-conditioned sludge was derived from the calculated release when each conditioner was applied individually. Observations from Figure 3a–f indicate that a synergistic enhancement of H_2_S emission occurred within the temperature range of 550~650 °C when CaO was combined with Fe_2_O_3_ (or FeCl_3_), with the actual emissions exceeding the theoretical predictions. This divergence intensified as the temperature rose. For example, at a 1:1 ratio of CaO to Fe_2_O_3_, the actual emission exceeded the theoretical predictions by 1.2% at 650 °C. This could be attributed to the sintering reaction between CaO and Fe_2_O_3_ at high temperatures, potentially leading to the formation of Ca-Fe compounds such as Ca_2_Fe_2_O_5_ [12]. As a result, the quantity of CaO available to react with sulfur is reduced, thereby diminishing its ability to immobilize H_2_S [22]. Similar patterns were detected when CaO and Fe_2_O_3_ were combined at ratios of 2:1 and 1:2, with the actual emissions at 650 °C being 0.7% and 1.7% greater than the theoretical values, respectively. This suggests that a higher concentration of Fe_2_O_3_ promotes the formation of Ca-Fe compounds, which subsequently impedes the inhibition of H_2_S release.

Furthermore, an examination of Figure 3a–f indicates that within the 350~450 °C temperature range, the composite of CaO and Fe_2_O_3_ displayed negligible synergistic action, while the combination of CaO and FeCl_3_ resulted in a synergistic suppression of H_2_S release, with actual emission levels being lower than the theoretical prediction. As depicted in Figure 2, which concerned the individual conditioning process, FeCl_3_ decomposed at higher temperatures to yield Fe_2_O_3_ and HCl. The formed HCl can react with inorganic or organic sulfur compounds in the sludge, thus facilitating the production of H_2_S. Consequently, the strong alkalinity of CaO, when combined with FeCl_3_, may neutralize the acidic conditions arising from the decomposition of FeCl_3_, thereby effectively reducing the emission of H_2_S.

In contrast to the combination of CaO with Fe-based conditioners, Figure 3g–i demonstrate that the composite of Fe_2_O_3_ and FeCl_3_ induced a synergistic promotion effect on H_2_S release between approximately 350 and 550 °C. This effect is attributed to the fact that at lower temperatures, both Fe_2_O_3_ and FeCl_3_ enhance the release of H_2_S, with FeCl_3_ having a particularly significant impact (as depicted in Figure 2). Notably, Figure 3i reveals that the combination of CaO and FeCl_3_ at a 1:2 ratio exhibited a more pronounced synergistic promotion effect compared to other composite ratios. This again indicates that the promotional effect on H_2_S release may originate from the HCl produced by the decomposition of FeCl_3_. At 650 °C, a synergistic inhibitory effect on H_2_S was observed, which intensified with an increased proportion of Fe_2_O_3_. Specifically, when Fe_2_O_3_ and FeCl_3_ were combined at ratios of 1:2, 1:1, and 2:1, the actual release was 0.3%, 0.6%, and 1.1% lower than the theoretical predictive value, respectively. It is possible that the oxidation reaction of organic sulfur by Fe_2_O_3_ competes with the reaction between HCl and organic sulfur, leading to a reduction in the negative impact of HCl.

**Figure 4:**
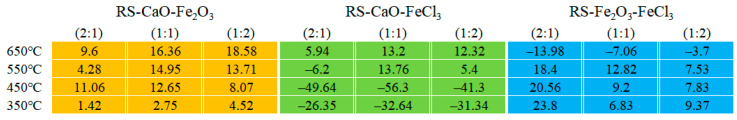
The synergistic effect of composite conditioners in suppressing the release of H_2_S during sludge pyrolysis.

The synergistic effects of composite conditioners on H_2_S release, as depicted in Figure 4, are primarily manifested as synergistic suppression within the 350~450 °C temperature range for the combination of CaO and FeCl_3_, and at 650 °C for the combination of Fe_2_O_3_ and FeCl_3_. The synergistic inhibitory effect observed with the composite of CaO and FeCl_3_, as inferred from Figure 3, is attributed to the strong alkalinity of CaO neutralizing the HCl generated by the decomposition of FeCl_3_. This neutralization effectively prevents HCl from engaging in reactions with inorganic or organic sulfur compounds presented in the sludge, thus suppressing the production of H_2_S. At 550 °C, only the RS-CaO-FeCl_3_ composite at a ratio of 2:1 exhibited a minor synergistic inhibitory effect on H_2_S release. However, as the proportion of FeCl_3_ increased, a synergistic promotion effect was observed, potentially due to the elevated Fe content facilitating sintering reactions that form Ca-Fe compounds. At 650 °C, the composite of Fe_2_O_3_ and FeCl_3_ alone demonstrated a synergistic inhibitory effect on H_2_S release, which may be attributed to the competitive reaction between the oxidation of organic sulfur by Fe_2_O_3_ and the reaction of HCl with organic sulfur at high temperatures, which could potentially reduce the negative impact of HCl. Despite the presence of Cl not being conducive to the suppression of H_2_S release, the sulfur-fixing capacity of Fe at high temperatures appears to override this effect. In conclusion, the selection of the composite of CaO and FeCl_3_ for the 350~450 °C range, the CaO and FeCl_3_ composite at a 2:1 ratio for 550 °C, and the Fe_2_O_3_ and FeCl_3_ composite for 650 °C was suggested to achieve a synergistic inhibitory effect on H_2_S release during sludge pyrolysis.

### 2.3. Effect of Conditioners on Sulfur Species in Char

#### 2.3.1. Effect of Single Conditioner on Sulfur Species

**Figure 5:**
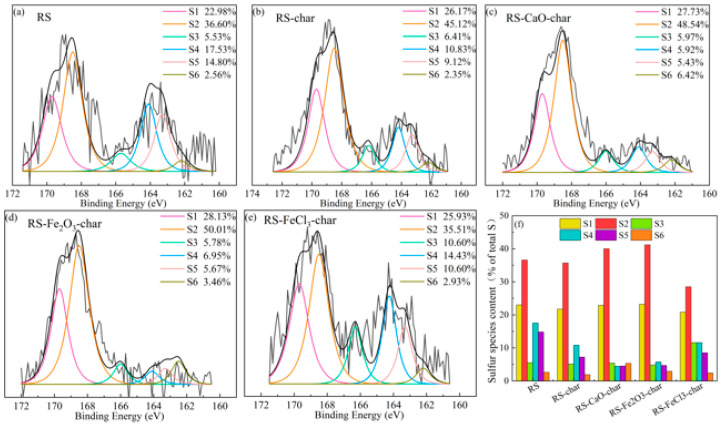
Distribution of sulfur-containing substances in sludge and pyrolysis char. (**a**) RS, (**b**) RS-char, (**c**) RS-CaO-char, (**d**) RS-Fe_2_O_3_-char, (**e**) RS-FeCl_3_-char, and (**f**) the content of sulfur-containing substances. (S1: inorganic sulfates; S2: sulfones; S3: sulfoxides; S4: aromatic sulfur; S5: aliphatic sulfur; S6: inorganic sulfides).

In order to investigate the mechanism of sulfur fixation in the char by various conditioners, considering that the inhibitory effect of the conditioner on H_2_S release is most pronounced at 650 °C, XPS was employed to examine the changes in sulfur-containing functional groups within the RS and the char obtained from pyrolysis at 650 °C. Figure 5a–e present the S 2p spectral profiles for both the sludge and the resultant pyrolysis char. Figure 5f displays the relative percentages of different sulfur species to the total sulfur content before pyrolysis. The sulfur in RS was distributed as inorganic sulfates (23.0%), sulfones (36.6%), sulfoxides (5.5%), aromatic sulfur (17.5%), aliphatic sulfur (14.8%), and inorganic sulfides (2.6%). Upon pyrolysis of RS, the levels of inorganic sulfates, sulfones, sulfoxides, and inorganic sulfides exhibited negligible variation (<1.3%), signifying their high stability. Conversely, the proportions of aliphatic and aromatic sulfur compounds were reduced to 7.2% and 10.8%, respectively, reflecting an overall decrease of 14.8%. Given that 10.0% of the sulfur was released as H_2_S during the pyrolysis of RS, as depicted in Figure 2, this suggests that H_2_S is predominantly derived from the decomposition of aliphatic and aromatic sulfur compounds [18].

In comparison to RS-char, the RS-CaO-char exhibited a reduction in the concentrations of aromatic and aliphatic sulfur by 6.4% and 2.7%, respectively. This reduction suggests that CaO has enhanced the decomposition or transformation of these organic sulfur species. Concurrently, there was an increase in the levels of inorganic sulfates, sulfones, and sulfoxides by 1.2%, 4.3%, and 0.3%, respectively. Upon closer examination of the chemical species, including aliphatic and aromatic sulfur (R_1_-S-R_2_), sulfoxides (R_1_-SO-R_2_), and sulfones (R_1_-SO_2_-R_2_), it was deduced that CaO acted as a catalyst, promoting the conversion of organic sulfur into more stable, oxidized forms. This conversion reduced the likelihood of these species being converted into gas-S during pyrolysis. Furthermore, the observed increase in inorganic sulfide content (3.4%) indicated that CaO may be involved in capturing gas-S.

Within the RS-Fe_2_O_3_-char, there were respective decreases of 5.1% and 2.5% in the levels of aromatic and aliphatic sulfur compounds, respectively, which aligns with the trend observed in the RS-CaO-char, albeit with a less pronounced effect. Concurrently, there was an increase of 1.5% in inorganic sulfates and a 5.5% increase in the concentrations of sulfones. Meanwhile, the concentration of sulfoxides declined by 0.3%. It is hypothesized that Fe_2_O_3_ shares a comparable role with CaO in facilitating the generation of oxidized sulfur species. Due to its more potent oxidative capacity, Fe_2_O_3_ was capable of yielding an increased quantity of sulfones [19]. Nevertheless, a modest increase of only 1.0% was observed in inorganic sulfide, which is below the 3.4% escalation achieved with CaO. On the one hand, this is because the conditioner has strong basicity that can capture acidic sulfur-containing gases to form inorganic sulfide, as indicated by the XRD analysis. However, Fe_2_O_3_ has a lower basicity compared to CaO, thus its ability to capture gas-S is relatively weaker.

Compared to RS-char, the levels of aliphatic and aromatic sulfur in RS-FeCl_3_-char increased by 1.3% and 1.2%, respectively. This indicates that FeCl_3_ suppressed their decomposition. Concurrently, there was a 7.2% reduction in sulfones, contrasting with a 6.5% increase in sulfoxides. This suggests that FeCl_3_ has a detrimental impact on the oxidation of organic sulfur, and its reductive properties may have prompted the conversion of sulfones to sulfoxides. The quantity of inorganic sulfides experienced a 0.5% increase. Considering that this increase in inorganic sulfides was not derived from the oxidation of other sulfur species, it is probable that FeCl_3_ captured gas-S and effectively sequestered the sulfur within the char.

#### 2.3.2. Synergistic Effect of Composite Conditioners on Sulfur Species

**Figure 6:**
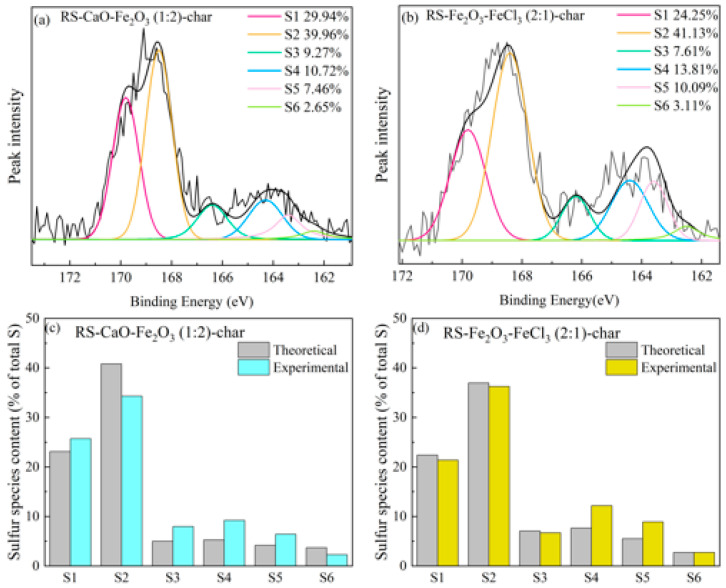
Distribution of sulfur-containing products in the pyrolysis char of composite-conditioned sludge. (**a**) The S 2p spectrum of RS-CaO-Fe_2_O_3_ (1:1)-char, (**b**) the S 2p spectrum of RS-Fe_2_O_3_-FeCl_3_ (2:1)-char, (**c**) the content of sulfur-containing substances of RS-CaO-Fe_2_O_3_ (1:1)-char, and (**d**) the content of sulfur-containing substances of RS-Fe_2_O_3_-FeCl_3_ (2:1)-char. (S1: inorganic sulfates; S2: sulfones; S3: sulfoxides; S4: aromatic sulfur; S5: aliphatic sulfur; and S6: inorganic sulfides).

To investigate the synergistic effects of composite conditioners, it has been observed that Ca-Fe conditioners exhibit a synergistic promotion effect at 650 °C, while Fe_2_O_3_-FeCl_3_ demonstrates a synergistic inhibition effect at the same temperature. The chars obtained from the pyrolysis of RS-CaO-Fe_2_O_3_ (1:2) and RS-Fe_2_O_3_-FeCl_3_ (2:1) at 650 °C were selected for analysis, considering their optimal synergistic inhibitory or promotive effects. These residues were subjected to XPS analysis, with the resulting S 2p peaks depicted in Figure 6a,b. The percentage composition of various sulfur species relative to the total sulfur content prior to pyrolysis is illustrated in Figure 6c,d.

In the case of the RS-CaO-Fe_2_O_3_ (1:2) composite, as depicted in Figure 6c, the measured sulfone content was observed to be 6.5% less than the predicted theoretical value. The levels of aromatic and aliphatic sulfur were 3.9% and 2.2% above the theoretical estimates, respectively. This suggests that the combination of CaO and Fe_2_O_3_ hinders the oxidation of aromatic and aliphatic sulfur into sulfones, which is detrimental to the reduction in H_2_S emissions. Moreover, the actual measured quantity of inorganic sulfides was 1.4% less than the expected value, indicating that the CaO and Fe_2_O_3_ composite has a reduced capability to capture gas-S. These findings align with the analysis shown in Figure 3, positing that at high temperatures, the interaction between CaO and Fe_2_O_3_, potentially forming Ca-Fe compounds, reduces their oxidative impact on organic sulfur compounds and their capacity to capture gas-S.

In the instance of the RS-Fe_2_O_3_-FeCl_3_ (2:1) composite, as shown in Figure 6d, the differences between the experimental and theoretical values for sulfones and sulfoxides were negligible, indicating that the combination of Fe_2_O_3_ and FeCl_3_ maintains a strong oxidative effect on the conversion of aromatic and aliphatic sulfur species into these compounds. Nonetheless, the measured values for aromatic and aliphatic sulfur were increased by 4.5% and 3.4%, respectively, when contrasted with the theoretical values. This corroborates the phenomenon observed in Figure 3g, where the actual release of H_2_S is subjugated to theoretical estimates, implying that the composite of Fe_2_O_3_ and FeCl_3_ suppresses the decomposition of aromatic and aliphatic sulfur species, thereby reducing H_2_S release. This is further supported by the analysis presented in Figure 4.

#### 2.3.3. Sulfur-Containing Mineral Components

**Figure 7:**
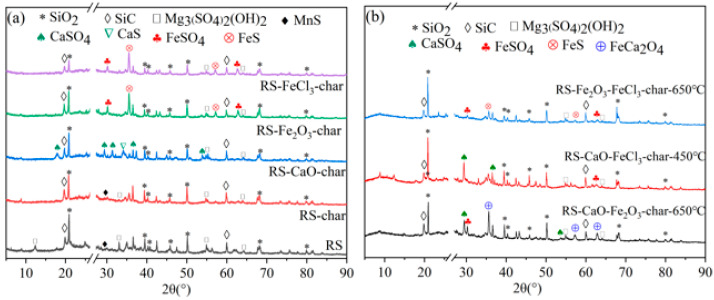
XRD spectrum of solid-phase products from sludge pyrolysis: (**a**) RS and singly conditioned sludge; (**b**) composite-conditioned sludge.

To further clarify whether the Ca-Fe composite conditioners sinter at high temperatures to produce Ca-Fe compounds, and to reveal the mechanism behind the increased presence of inorganic sulfates and inorganic sulfides in the char as depicted in Figure 5, an analysis of the mineral components of the pyrolysis samples was conducted using XRD. Figure 7a displays the XRD spectra of the sludge and char obtained at 650 °C. A comparison between RS-char and RS-CaO-char revealed distinct peaks corresponding to CaSO_4_ and CaS in RS-CaO-char, which were absent in RS-char. This observation confirms that CaO facilitates the retention of sulfur as inorganic sulfates and inorganic sulfides within the char. As a result, the sulfur content in char (char-S) increases, while the sulfur in gaseous phase (gas-S) decreases, in accordance with the data presented in Table 1. Additionally, the identified forms of inorganic sulfates and inorganic sulfides, specifically CaSO_4_ and CaS, are believed to be the consequence of emitted SO_2_ and H_2_S being captured by CaO. Similarly, the presence of FeSO_4_ and FeS in RS-Fe_2_O_3_ and RS-FeCl_3_ indicates that Fe-based conditioners exhibit a sulfur-fixing reaction similar to that of CaO. Both Fe-based and Ca-based conditioners effectively immobilize sulfur by capturing gas-S and forming inorganic sulfates and sulfides within the char, as depicted in Figure 5.

The XRD spectra of the solid-phase products resulting from the pyrolysis of composite-conditioned sludge are depicted in Figure 7b. There are peaks corresponding to CaSO_4_, FeSO_4_, and FeS within the char of composite-conditioned sludge, identical to those observed in Figure 7a. This indicates that the composite conditioners are capable of sulfur fixation through the direct capture of gas-S. However, it is noteworthy that when CaO was combined with Fe_2_O_3_, the presence of FeCa_2_O_4_ substance peaks was detected in the char of RS-CaO-Fe_2_O_3_ obtained at 650 °C. These peaks were not observed in the char of RS-CaO-FeCl_3_ obtained at 450 °C, nor in the char of the singly conditioned sludge (as shown in Figure 7a). This finding confirms that the reaction between Ca-based and Fe-based conditioners at high temperatures leads to the formation of Ca-Fe compounds. As a result of this reaction, their capacity to capture gaseous sulfur is reduced, which in turn contributes to an increased release of H_2_S.

## 3. Materials and Methods

### 3.1. Sample Preparation

In this study, the RS was collected from the Xinkaipu wastewater treatment plant located in Changsha, Hunan. The RS was first dried at 105 °C until a constant weight was achieved. Subsequently, it was ground and sieved to obtain a particle size of ≤80 mesh [23]. The processed samples were then stored in a light-protected environment for subsequent use. In accordance with the standards “GB/T28731-2012” and “GB/T31391-2015” [24,25], both the proximate and ultimate analyses (Elementar Vario EL, Hanau, Germany) of the RS were conducted, respectively, with the results detailed in Table 2.

The reagents CaO, Fe_2_O_3_, and FeCl_3_ utilized in this study were all obtained from Sinopharm Chemical Reagent Co., Ltd. (Shanghai, China). The conditioners were mixed with the RS in a dry state [26], using a mass ratio of 10:1 for RS to conditioner [15]. The sludge samples amended with CaO, Fe_2_O_3_, and FeCl_3_ were designated as RS-CaO, RS-Fe_2_O_3_, and RS-FeCl_3_, respectively. The composite conditioners were prepared at mixing ratios of 1:1, 2:1, and 1:2. The samples of sludge conditioned with the composite additives were denoted as RS-A-B (R), where ‘A’ and ‘B’ represent the respective conditioners, and ‘R’ indicates the mixing ratio. For example, RS-CaO-Fe_2_O_3_ (1:1) referred to the sludge treated with a 1:1 mixture of CaO and Fe_2_O_3_ conditioner. The resulting chars from the pyrolysis of different sludge samples are abbreviated as X-char, with X representing the specific sludge type.

### 3.2. Pyrolysis Experiment

Sludge pyrolysis experiments were performed using a horizontal tube furnace in a controlled experimental setup, which is consistent with that of our group’s previous study [27,28]. Each trial involved the pyrolysis of 1 g of sludge, carried out under a nitrogen atmosphere with a flow rate of 200 NmL/min. The experiments were conducted over a temperature range from 350 to 700 °C, with each run lasting for a period of 20 min [14]. After the tubular furnace reached the preset temperature for the pyrolysis reaction, the sample was quickly inserted into the center of the heating tube. Gaseous products were collected using gas collection bags. After the reaction, the char was cooled to room temperature under a nitrogen atmosphere before being collected for further analysis [29].

### 3.3. Analytic Methods

The content of sulfur in both the RS and the derived pyrolysis char was determined using an elemental analyzer. The presence of inorganic crystalline sulfur-containing phases in the RS and char was investigated using an X-ray diffractometer (XRD, Rigaku Ultima IV, CuKa, Japan) with scanning angles ranging from 5° to 90°. The XRD data were analyzed using Jade 6 software, with reference to the PDF-2 (Powder Diffraction File) database. Additionally, the chemical speciation of sulfur in the RS and char was analyzed using an X-ray photoelectron spectrometer (XPS, Thermo Scientific K-Alpha, Waltham, MA, USA) with an aluminum anode operating at 12 kV and 6 mA. The sulfur species were identified based on their binding energies and classified into inorganic sulfates (170 ± 0.3 eV), sulfones (168.2 ± 0.3 eV), sulfoxides (166 ± 0.3 eV), aromatic sulfur (164.1 ± 0.3 eV), aliphatic sulfur (163.3 ± 0.3 eV), and inorganic sulfides (162.2 ± 0.3 eV) [14,30]. The quantification of sulfur-containing gases was performed using a gas chromatograph (GC, HC-3, Wuhan, China). This GC system incorporates a dual-column arrangement designed to efficiently separate a spectrum of sulfur-containing gaseous compounds. The detection capabilities are highly sensitive, with limits as low as 0.1 ppm, facilitated by a flame photometric detector. Prior to ignition, the outlet pressures of the hydrogen (H_2_) and oxygen (O_2_) regulators were set to 0.1 MPa. Additionally, the H_2_ and O_2_ pressures were adjusted on the instrument panel to 0.03 MPa. Once the column and detector reached the necessary temperatures, ignition could proceed. The set temperatures for columns 1 and 2 were 92.5 °C and 46.8 °C, respectively.

### 3.4. Calculation Method

The percentage of different forms of sulfur in the solid phase to total sulfur before pyrolysis was denoted as *P_S from char_*, and calculated using Equation (2).

(2) $$P_{S\;from\;char}=\frac{S_{C}*S_{A}}{S_{total}}$$

where *S_C_* was the total sulfur content in char; *S_A_* was the percentage of the peak area corresponding to the sulfur compound ‘A’ in the XPS spectrum, relative to the total peak area; *S_total_* was the total sulfur content in the sludge before pyrolysis.

The percentage of sulfur in various forms in the gas phase relative to the total sulfur content before pyrolysis, was denoted as *P_S from gas_*. This value was calculated using Equation (3), with H_2_S serving as an example:

(3) $$P_{S\;from\;H_{2}S}=M_{H_{2}S}*\frac{C_{H_{2}S}*V}{S_{total}*1000}$$

where *P_S from H2S_* is the percentage of H_2_S relative to the total sulfur content before pyrolysis; *M_H2S_* is the ratio of the molar mass of sulfur atoms to the molar mass of H_2_S molecules; *C_H2S_* is the concentration of H_2_S in the gas phase; and *V* is the volume of H_2_S.

The theoretical yield of sulfur-containing products in both gas and solid phases, derived from the pyrolysis of composite-conditioned sludge, was denoted as *Y_Theo_*, and calculated using Equation (4), with the theoretical release of H_2_S serving as an example:

(4) $$Y_{Theo}=\frac{x_{1}Y_{1}+x_{2}Y_{2}}{x_{1}+x_{2}}$$

where *Y*_1_ and *Y*_2_ are the release amounts of H_2_S when conditioners 1 and 2 were used individually to treat the sludge, respectively; *x*_1_ and *x*_2_ are the percentages of conditioners 1 and 2 in the composite conditioner, respectively.

The synergistic effect value, which quantified the composite conditioner’s ability to suppress the release of H_2_S during the sludge pyrolysis, was calculated using Equation (5).

(5) $$s_{ynergistic\;\;effect}=\frac{Y_{Exp}-Y_{Theo}}{Y_{Theo}}*100$$

where *Y_Exp_* and *Y_Theo_* correspond to the actual and theoretical release amounts of H_2_S, respectively. A negative *S_ynergistic effect_* value indicates that the combination of the two conditioners induced a synergistic suppression effect on the release of H_2_S during pyrolysis. Conversely, a positive *S_ynergistic effect_* value suggests that the combination facilitated the emission of H_2_S.

## 4. Conclusions

This study clarified the effects of three conditioners (CaO, Fe_2_O_3_, and FeCl_3_) on the sulfur distribution within the products of sludge pyrolysis. By conducting a comparative analysis of the theoretical and experimental values of sulfur-containing substances in the products, the synergistic effects of composite conditioners and their influence on the emission of sulfur-containing gases were explored. The derived conclusions are as follows:

(1) The release of H_2_S was suppressed by CaO, Fe_2_O_3_, and FeCl_3_ by 35.8%, 23.2%, and 9.1% at 650 °C, respectively. CaO mitigated gas-S release by promoting the transformation of organic sulfur into more stable oxidized forms, and by capturing gas-S. Fe_2_O_3_ demonstrated enhanced oxidizing capabilities, while FeCl_3_ inhibited the decomposition of organic sulfur and captured gas-S at high temperatures.
(2) Utilizing a composite of CaO and FeCl_3_ within the temperature range from 350 to 450 °C, along with a composite of Fe_2_O_3_ and FeCl_3_ at 650 °C, can synergistically suppress the H_2_S release. Conversely, between 350 °C and 550 °C, the combination of Fe_2_O_3_ with FeCl_3_, and between approximately 550 and 650 °C, the composite of Ca-based and Fe-based conditioners, demonstrate a synergistic enhancement effect on the release of H_2_S.
(3) In composite conditioning, the strong alkalinity of CaO neutralizes HCl, an acidic gas generated by the decomposition of FeCl_3_. This neutralization prevents HCl from interacting with inorganic or organic sulfides in the sludge, thus synergistically inhibiting H_2_S release. At 450 °C, this synergistic effect inhibits H_2_S release by 56.3%. Nonetheless, the formation of FeCa_2_O_4_ above 550 °C, reduces the sulfur-fixing capacity of the composite conditioners, and at 650 °C, it synergistically promotes the release of H_2_S by 26.3%.

Future research should focus on examining the impact of complex conditioners on the kinetics of pyrolysis and delve into the molecular level to explore the mechanism of sulfur fixation. To enhance the optimization of the dewatering, conditioning, and pyrolysis process, an economic assessment and comparative analysis of various pathways will be essential.

## Figures and Tables

**Table 1:** The distribution of sulfur among various products.

	Char-S (%)	Gas-S (%)	Tas-S (%)
RS-700	79.1	15.1	-
RS-CaO	90.6	7.0	-
RS-Fe_2_O_3_	89.8	6.9	-
RS-FeCl_3_	85.5	9.9	-

**Table 2:** Proximate and ultimate analysis of RS.

Samples	Proximate Analysis (wt.%) ^a^			Ultimate Analysis (wt.%) ^a^				
	Volatile Matter	Ash	Fixed Carbon	C	H	N	S	O ^b^
RS	31.2	64.7	4.1	15.88	2.64	2.52	1.11	13.15

^a^ dry ash basis ^b^ calculated by difference.

## Data Availability

Data are available upon request.
